# Co-production of an e-resource to help women who have experienced childhood sexual abuse prepare for pregnancy, birth, and parenthood

**DOI:** 10.1186/s12884-020-03515-5

**Published:** 2021-01-07

**Authors:** Elsa Montgomery, Julia S. Seng, Yan-Shing Chang

**Affiliations:** 1 Florence Nightingale Faculty of Nursing, Midwifery and Palliative Care, King’s College London, 57 Waterloo Road, London, SE1 8WA UK; 2 School of Nursing, University of Michigan, 400 North Ingalls Building, Ann Arbor, MI 48109-5482 USA

**Keywords:** Childhood sexual abuse, Pregnancy, Birth, Parenthood, Co-production, E-resource

## Abstract

**Background:**

This paper reports the development of a co-produced e-resource to support those who have experienced childhood sexual abuse through pregnancy, birth, and parenthood. These are times of major transition for any woman but can present particular challenges for those who have experienced childhood sexual abuse. Re-traumatisation during the perinatal period is common and can occur in ways that may not be anticipated by those involved. Survivors often do not disclose their abuse and the childbearing journey can be lonely.

**Methods:**

The work was conducted in collaboration with The Survivors Trust and in keeping with the Survivor’s Charter. A participatory approach was used. There were two phases: the generation of new qualitative data and development of the resource. To encourage participation from this hidden population, data were collected by a variety of means including focus groups, telephone interviews and an on-line survey. Survivors who had children and those who hoped to one day participated. Resource development was facilitated by two workshops and email feedback.

**Results:**

Overall, 37 women participated, all of whom were positive about development of the resource. Although many issues identified during data collection were specific to the participants’ history of abuse other areas of concern would be relevant for any woman contemplating the journey to parenthood. Women often assumed that they were alone in their concerns and were reassured to discover that others shared their experiences. The final resource is hosted on The Survivors Trust Website and is accessible from all electronic devices. It follows the journey from deciding to have a baby, pregnancy, labour, birth, and the postnatal period through to parenthood. Links are provided to further information and sources of support. The process of developing the resource used trauma-informed principles and it speaks with women’s words in a peer-to-peer voice.

**Conclusions:**

This paper describes the development of an innovative and accessible e-resource that is based on the words and experiences of survivors of childhood sexual abuse. It recognises the importance of control and feeling safe and aims to empower those who use the resource as they embark on pregnancy, birth, and parenthood.

## Background

Childhood sexual abuse (CSA) has been described as one of the most serious public health issues facing society [1]. Determining prevalence is very difficult [2] but a recent worldwide meta-analysis including women from 16 different countries, discovered an overall pooled CSA prevalence of 24% [3]. Although there was no difference in rates between developed and developing countries, there was considerable geographical variation (highest in North America at 32% and lowest in Africa at 8%). The authors suggest this may be due to bias as only one study from Africa was eligible for inclusion. No studies from the United Kingdom (UK) were included in this meta-analysis, but the rate in Europe was found to be 17%. It is estimated that there are about 1.3 million children living in the UK who will have experienced sexual abuse by the time they reach the age of 18 [4]. As only one in eight of these children come to the attention of statutory bodies, seven out of eight potentially end up hidden as adults. CSA ‘casts a long shadow’ [4]. Significant physical and psychological morbidity is recognised in this population [5] and consequently survivors are heavy users of health services. We use the term ‘survivor’ as useful shorthand. We recognise that not all women who have experienced childhood sexual abuse find this a relevant or helpful term.

Pregnancy and birth are times of biological, psychological, and sociological transition for any woman but can present particular challenges for those who have experienced CSA. Aspects of pregnancy, perinatal care, birth, or early parenting can be reminders of past trauma and ‘trigger’ the ‘re-experiencing’ of sensations, emotions, and posttraumatic stress reactions [6]. Survivors report ‘re-enactment’ of their abuse during pregnancy and birth in ways that they may not have anticipated and that may be very perplexing at the time [7]. They have indicated that it would have helped them to know that their experiences are shared by other survivors. Many survivors report guilt and shame in relation to their childhood history and although they feel scared and alone during their childbearing journey, they dare not ask for help from those providing health care because they fear judgment [8]. Consequently, few disclose their abuse to those caring for them.

Silence was an all-pervading theme in previous work by the first author into the maternity care experiences of women who have experienced CSA [9]. The participants in her narrative study spoke with eloquence and their words were very powerful. Based on this research, we decided that one way to address the challenges of guilt, shame and silence for people who have experienced CSA as they contemplate pregnancy, birth and parenthood would be to create an e- resource using these words. Initial conversations with survivors and those who support them in the voluntary sector confirmed that such a resource would be helpful.

## Methods

### Aim

The aim of this study was to co-produce an e-resource to help prepare women who have experienced CSA for pregnancy, birth, and early parenthood, grounded in their experiences and concerns. It was conducted in collaboration with The Survivors Trust (TST), which is the largest umbrella agency for specialist rape and sexual abuse services in the UK and has 129 member agencies based in the UK and Ireland (http://thesurvivorstrust.org/about-tst/).

### Setting and Design

The work was conducted in London UK, and ethical approval was gained from King’s College London Psychiatry, Nursing and Midwifery Research Ethics Subcommittee. As power, control and disempowerment are such important issues for those who have experienced CSA [10], ‘an environment of collaboration’ in keeping with the Survivor’s Charter [11] was of paramount importance in our work. Our approach was therefore guided by the principles of participatory research but focused on elements required to realise our goal of co-producing the resource [12]. Empowerment was a key driver in the research process [13]. The study was overseen by a Project Advisory Group all of whom had been co-applicants on our funding bid. Members of the group (*n* = 4, three of whom are survivors) included those with personal experience of CSA, people with relevant research and professional expertise, plus the research team from King’s College London. Members of the Advisory Group with personal experience of CSA commented on drafts of study documents.

### Data collection

Co-production of the resource involved two main phases:

- Phase 1: Generating new data with women who have experienced CSA
  - ∘ Phase 1A explored what those who have had children wished they had known before they embarked on pregnancy and birth. The extent to which they felt their abuse impacted the experience and helpful coping strategies were discussed.
  - ∘ Phase 1B explored the hopes, fears, and questions of those who wished to have a baby one day.

Participants engaged with the project knowing they were contributing to developing a resource to help other survivors and thus framed their experiences accordingly.

- Phase 2: Development of the resource: an iterative process with the purpose of discussing specific ideas for the resource formulated following Phase 1, creating it, and doing initial evaluation.

All those who participated in the study were members of organisations belonging to TST, were aged 18 years or above and were able to speak and read English. They had been sexually abused in childhood and either had experience of pregnancy and/or birth or were contemplating having a baby. Data in Phase 1 were initially collected via two focus groups; recruitment was conducted through TST. Information about the study, an invitation to participate, a participant information sheet, and a reply slip were sent by TST to their member organisations who then cascaded all the information to their own service users. Anyone who was interested contacted the research team and mutually convenient dates for the focus groups were organised.

Consent was gained prior to the start of each group and a leaflet outlining sources of support was provided to the participants at the end. All participants were given a £20 high street voucher as a token of our appreciation for each event they attended in person (i.e. during Phase 1 and 2). Travel and childcare expenses were reimbursed. Although it has been suggested that focus groups may not be a good way to collect data on sensitive or challenging subjects [14] there are benefits which made them suitable for this study. They are frequently used in participatory research in which they are important in the creation of a “communicative space” [15]. The interaction among participants that is key in focus groups [16] leads to richness of data that may not be possible from individual interviews [17]. The number of people present tends to shift the locus of control away from the researcher to the participants [16]. The questions used to prompt discussion had been discussed with the Advisory Group and were crafted to facilitate open discussion. As control is crucial to ‘feeling safe’ for those who have experienced CSA [10], these factors were an important part of our approach. Althouth the ideal number for a focus group is five to eight participants, they may be conducted with as few as three [18] and a smaller group may be less intimidating when the subject is sensitive.

Survivors of CSA are a silent, hidden, population [8] whose involvement in research can impact coping mechanisms [11]. We had intended to recruit participants for four focus groups of up to eight people each, but despite initial interest and reminder invitations, only six women attended overall (*n* = 3 for groups in phase 1A and 1B). One of our Advisory Group members who had children participated in phase 1A and another who had not yet had children participated in phase 1B. Groups met once for approximately 2 h. Refreshments were provided.

Following consultation with our Advisory Group and with relevant approvals, our data collection strategy was consequently revised to offer more options for participation. Using the same recruitment strategy, further data were collected via telephone interviews (*n* = 2) and an on-line survey with open-ended questions that was accessed by 60 participants, 29 of whom provided responses.

Focus groups and interviews were digitally audio-recorded and transcribed verbatim by a transcription company that had signed a confidentiality agreement. Transcripts were checked for accuracy against the recordings and anonymised by the first author. Data from the focus groups, interviews and open-ended questions from the survey were analysed using an inductive approach outlined by Braun and Clarke [19]. Initially data analysis was conducted independently by two of the research team. Both had been present at the focus groups and one had conducted the interviews as well. The first stage involved familiarisation through reading and re-reading. The second stage involved data-driven coding and was managed with NVivo 11. Codes were then organised into sub-themes which were discussed and agreed by the two researchers. Sub-themes were reviewed and organised into overall themes. This stage of analysis was guided by the goal of resource production.

Phase 2 involved development of the resource and was driven by data from Phase 1. An initial outline plan of the resource was written by the research team and discussed at an Advisory Group Meeting. More detail was then added to the plan incorporating feedback from the Advisory Group. All participants from Phase 1 were invited to a workshop during which a presentation of the planned resource was discussed. Six women attended and provided detailed feedback and suggestions for extra content. Participants who were unable to attend in person were sent the outline plan by email and five provided feedback. The plan was also sent to members of the Project Advisory Group for further comment.

Feedback was incorporated and a prototype of the resource was produced. More specific detail is provided below, but in summary, we worked with film production company ‘Jmotion’ to create short films and animations of data from the previously conducted narrative study [7–9]. Learning technologists from King’s College London took the updated presentation from Workshop 1 and built the prototype e-resource integrating quotations from both data collected during Phase 1 and the narrative study. They also incorporated the videos. Four women were available for Workshop 2 which provided an opportunity for participants to test the resource. A link to the resource was sent to members of the Advisory Group, including JSS, for feedback. Other than that, the resource was not shared with those who could not attend in person at this stage to avoid wider circulation before the official launch.

## Findings

The total number of participants contributing to development of the resource was 37. Twenty-four had children and 13 hoped to in the future. Although the age of participants ranged from 20 to 68, 65% were in their twenties and thirties. Most participants were from a white ethnic background (*n* = 33). The other women described their ethnic group as Black (*n* = 1), Asian (*n* = 1), Mixed (*n* = 1) and other (*n* = 1). Eighty one percent (*n* = 30) of the participants were graduates as defined by the Office for National Statistics (those people who have left education with qualifications above A level standard).

Participants were overwhelmingly in support of our work to help women who have experienced CSA prepare for pregnancy, birth, and parenthood. Those who were not yet parents had some anxiety about how they would cope with the physical and emotional aspects of becoming a parent. Those who had experienced pregnancy and birth had not always appreciated the extent their childhood abuse would affect them. For clarity, as the main aim of this study was the co-production of a resource, findings from Phase 2 are presented first.

### Development of the resource

The outline content for the resource was generally very well received, and women appreciated being involved in its development:

*First of all I think this is an excellent resource and I'm so excited about developing it. You have covered so many important points it will be such a valuable resource for so many.* (Feedback via email)

The contribution that survivors were able to make for the benefit of others was also appreciated:

*It is really encouraging to see how much thought and consideration has gone in to putting this together, with a clear steer from survivors for survivors. It is indeed a privilege to take part and help shape the information that will help other survivors prepare for their pregnancy journey.* (Feedback via email)

The overall approach taken in structuring the resource was endorsed by those providing feedback. Sections take women on the journey from deciding whether to have a baby, through pregnancy, birth and becoming a parent. We responded to input that the topic is difficult and that some of the words are hard to hear by creating short sections. Users can choose when, where and how they access them. In its final form, there is a menu providing links to each section which facilitate choice and control. Further resources and sources of support are included at the end of the resource.

**Fig. 1:**
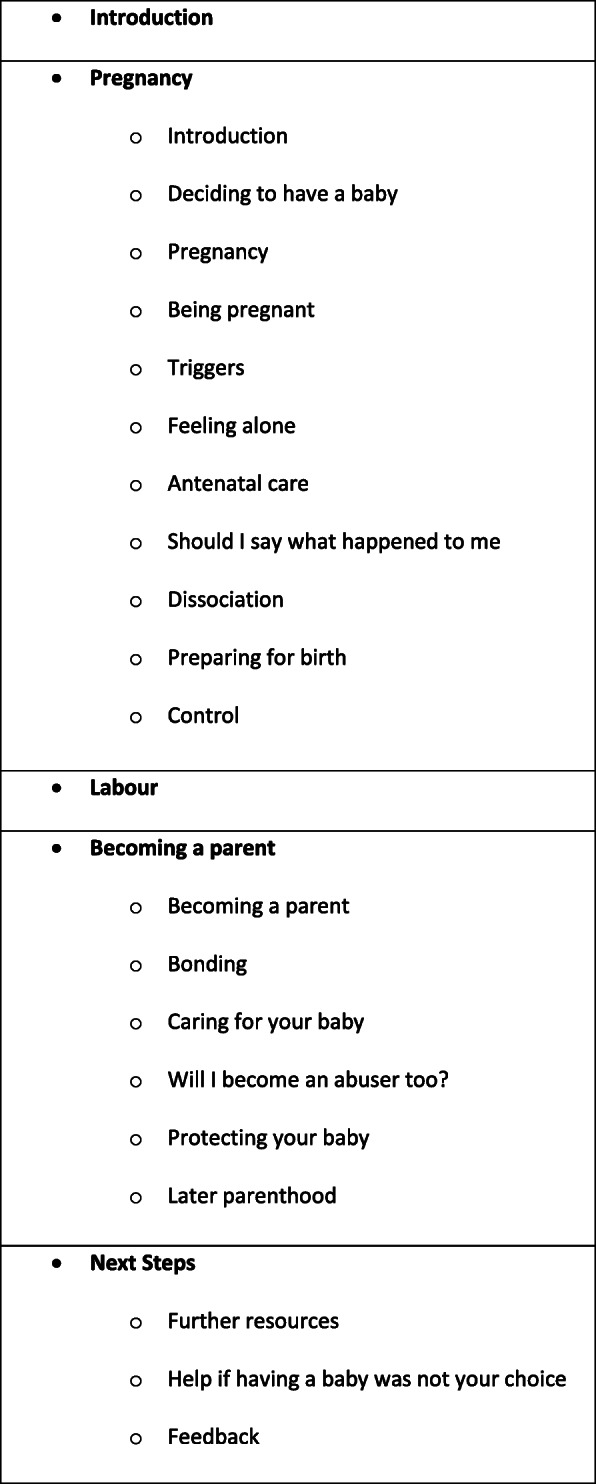
Sections in resource

Participants in this phase shared their individual experiences of issues mentioned in the outline plan and highlighted detailed information they thought would be helpful content. Many of the suggestions for additional content involved information that was not specific to a population of women who have experienced CSA but would be important in helping women to know what to expect and therefore feel safe and in control. Links to extra resources and websites for both general information (e.g. National Health Service (NHS) Information, a source of information on many aspects of health, including pregnancy – https://www.nhs.uk/conditions/pregancy-and-baby/what-happens-during-labour-and-birth) and subject-specific organisations (e.g. First Person Plural, for those with Dissociative Identity Disorder - https://www.firstpersonplural.org.uk) were included in the relevant sections. In response to feedback from members of the Advisory Group, content for women who were struggling to get pregnant and recognition that some may not have had a choice about being pregnant was included. We also added a section on ‘Later Parenthood’. Sections in the final resource are shown in Fig. 1.

Drawing on the feedback received, detailed content was developed for each section. Rich qualitative data were integrated into the e-resource directly; voices of the women who have shared their experiences are heard though quotes, films, and animation. Issues about care that emerged in the data were incorporated as suggestions, advice, or stories from survivors to survivors, to empower and inspire action on important matters. These include choice, the right to be informed and the right to say ‘no’.

Participants in workshop 2 viewed and evaluated the prototype. They completed an anonymous evaluation form, and all rated the resource as excellent. There was one suggestion for a slight change of wording in the section on infant feeding and the content was amended accordingly. Those who had experienced pregnancy and birth recognised their own experiences reflected in the resource and appreciated its candour:

*The resource covers so much and is excellently well put together. It is clear that it has been done with empathy and compassion and has not shielded the unpleasant and uncomfortable reality for some pregnant women or women planning to have children who have been sexually abused.* (Workshop Participant)

A range of views, opinions, and experience was expressed during data collection in Phase 1. This range was reflected in the resource, an aspect that was welcomed:

*Very relatable. The variety of feelings and experiences by survivors who have been at various stages of pregnancy. There was no right or wrong way to feel and allows all feelings to be validated.* (Workshop Participant)

However, we did not always get it right and a reminder during Phase 1 of how difficult some of those with a history of CSA find the word ‘survivor’ was a timely warning of the need for care with language.

### Experiences, hopes and fears of women who have experienced CSA

As it was important that the final resource reflected the needs of those it is intended to help, the initial stages involved exploring what survivors who have been through pregnancy and birth wished they had known at the time and the concerns of those who hoped to become parents one day. This exploration provided rich, insightful data that guided the content of the resource. Women often assumed that they were alone in their concerns and were reassured to discover that others shared their experiences. The following sections illustrate the main issues identified that were relevant to the resource. These are summarised in Table 1.

**Table 1:** Themes and sub-themes

Theme	Sub-themes
Pregnancy	Feelings about being pregnant
	Damage
	Expectations
	Public property
Re-living abuse	Triggers & flashbacks
	Loss of control
	Relevance of being a survivor
	Care without consent
Birth	Giving birth
	Labour
	Importance of continuity
Dissociation	Closing down
	Examinations
Disclosure	Asking the question
	Should I tell?
Parenthood	Becoming a parent
	Protecting the baby
	Being a good mother
	Caring for the baby
	Infant feeding

### Pregnancy

There were diverse responses to discovering the pregnancy as illustrated by the following comments from women:

*A mixture of delight, excitement and utter fear …* (Parent, survey)

*Worried, tired, exhausted, alone, ugly, fat, unloved, uncared for, little support.* (Parent, survey)

Pregnancy was a terrifying prospect for some of the participants even if it was what they wanted. There was uncertainty among those contemplating having a baby about whether their body would be capable of childbirth and even if their abuse might hurt the baby in some way:

*But there’s a fear isn’t there of is your body capable … is the abuse going to damage your child during pregnancy* (Non-parent, focus group)

Fear of physical damage from the abuse was a common concern. For some this fear reflected negative views women held about themselves; for others it was based on information from healthcare professionals. One woman was ‘overwhelmed’ to find she was pregnant and relieved to discover that she was able to have children:

*I was so emotional. I didn't expect to get pregnant as I was abused and raped for 10 years as a child on a daily basis. I thought because my perpetrator hurt me and I thought he damaged me so I was unable to have children.* (Parent, survey)

Another had been told by doctors following two pregnancies that were a result of rape, that CSA had caused too much damage for another baby to be possible. When she later became pregnant by her husband, her experience ‘was terrifying and nerve wracking’ (Parent, survey) even though she had been ‘elated’ about the pregnancy that produced her first child. There were complications for this woman because of the damage, but at the time of the survey, her child was a ‘happy and healthy 10-year-old’. Conversely, another woman wondered if giving birth would ‘be [her] rebirth’ (Non-parent, survey).

Experiences of being pregnant were also diverse and complex. One woman, despite having problems with body image, described how she loved being pregnant and felt very connected to all her babies during pregnancy. Others had very different experiences, as the quote below illustrates:

*I really hated being pregnant, I really hated it. And that in itself was really difficult because … you're kind of having check-ups and like the scans and you're going to the midwife all the time and people wanting to look at your stomach and measuring it and listening to it and kind of talk about you and had this expectation that you were going to react in a certain way* (Parent, interview)

Part of the challenge of both the prospect and reality of pregnancy for women who have experienced CSA is the lack of control and bodily autonomy it entails:

*During all of my pregnancies I have felt at times a complete loss of control and personal body autonomy. I’m not sure if this is usual or a result of my rapes.* (Parent, survey)

The ambivalence this created for women also brought feelings of guilt. One parent summed her situation up like this:

*Because I guess, kind of, surviving abuse, you kind of have to reclaim your body and your physicality and yourself and now you have to let go of it again for somebody else because you have to do all these things for the sake of the baby. So, kind of, if the baby wasn’t moving, I didn’t want to go and have to get looked at and tested but it’s just a real, kind of, it’s a real battle between your desire to want to be left and your body to be left alone and also a responsibility to your baby that you need to protect, so it was really difficult …* (Parent, interview)

Women perceived that lack of control arose both from the pregnancy itself and from the need to allow other people access to their body. Sometimes that access was unsolicited and unexpected. One woman recounted how she seemed to become public property when pregnant:

*People in the street (especially men) sometimes rubbed my bump without asking. This felt like a huge violation.* (Parent, survey)

Loss of control was mentioned in a number of contexts and was one of the ways through which women re-lived their abuse during pregnancy, birth, and early parenthood.

### Re-living abuse

As we have already heard, giving their bodies over to someone else (the baby) during pregnancy can remind women of their abuse. Women reported being transported back to their abuse in many other ways too. Anything from strong smells (e.g. aftershave or perfume), to changing a nappy were mentioned. Loss of control was particularly reminiscent of the experience of CSA and very disempowering:

*I would say not having control and being told what to do, how to do it …* (Parent, focus group)

Being subject to care that was ‘just done to you’ without consent (Parent, interview) was triggering; active involvement and being able to change plans was important.

The individual nature of triggers was recognised, but also the benefit of knowing about potential triggers:

*I wish I’d have known the type of triggers.* (Parent, focus group)

Concern was expressed by women who had not had children about the potential for triggers and flashbacks during pregnancy and birth. They were keen to discover if there were aspects of the journey that were particularly challenging and whether these had been unexpected for women who had given birth. This experience was confirmed by parents:

*It is sometimes the weird things or the unexpected that will cause you to be triggered.* (Parent, survey)

Just the mention of something could ‘open a can of worms’ (Parent, interview). Other triggering experiences were more predictable. Certain positions, especially those required during examinations could be challenging for women. They recognised that these examinations might be necessary but felt that an explanation of what was about to happen, and consideration of possible alternatives would help. Situations were particularly difficult if there seemed to be a power imbalance:

*If I feel like someone’s trying to overpower me with their position or their voice or their presence … it does cause a lot of triggers in that moment and in that situation.* (Parent, focus group)

Although the experience of birth was triggering for some women, it was often staff intervention as much as the process of birth itself that caused difficulty:

*… when it was time to push her out my legs clamped shut and I literally could not open them. I was so frightened and probably so triggered that I just could not open them. In the end I had two midwives pulling my legs apart in order to make enough space for her to come out. That was really awful and again quite triggering for me as having my legs pulled apart was something I had experienced during my abuse.* (Parent, survey)

Another respondent reported a situation that any woman would find difficult; a rush to theatre for trial of vaginal birth which potentially could have proceeded to an emergency Caesarean Section. However, for that woman, onlooking theatre staff were particularly reminiscent of an abusive situation:

*Then I was rushed into theatre for an emergency section. They said that if I could push her out, we should try that first. So I did! But, it was incredibly triggering for me, my legs were in stirrups and I couldn't move very easily, there were two midwives who were close, but all the theatre staff were around the edge watching (very similar to how it had been in one of my abuse situations).* (Parent, survey)

Traumatising birth experiences were common but not reported by all participants.

### Birth

Attitudes to birth varied. At one end of the spectrum labour was viewed as an interesting physical challenge:

*… so interestingly, I was quite looking forward to the labour in terms of before it, because I was quite interested in the physical challenge and how my body would respond and how I’d cope with it.* (Parent, interview)

At the other end, it was something to be feared:

*… and the actual giving birth and the idea of pain generally just really quite frightens me. I'm always asking people if I know someone who’s given birth I'm always like ‘how much did it hurt then’ …* (Non-parent, focus group)

Women who had experienced birth valued the presence of someone with whom they had a supportive relationship during labour. Sometimes their partner provided the necessary support:

*… and actually in that point my partner was really – you're really vulnerable and so my partner was really kind of supporting me, the labour itself was actually, was OK …* (Parent, interview)

Others valued the presence of supporters such as a doula (a woman without the formal training of a healthcare professional who provides support to women during childbirth). Continuity of carer was key for many women and a relationship with the midwife made a huge difference, even if things did not go to plan:

*But my midwife, she was with me, she was my community midwife and she was with me all through the delivery with my first son … although it was in hospital and that wasn’t what I wanted, it was the absolute best delivery I could have had.* (Parent, interview)

More medicalised births were often challenging for women. The position they had to adopt on a bed was one aspect, but often there were additional people present, which was also difficult:

*And I didn’t want to be on the bed with people around me and looking at me anyway but being in that position with – it was just awful, it was just awful.* (Parent, interview)

The attitude of care givers was a key factor in how women experienced labour and birth. Insensitive care was particularly traumatising because it brought back memories of women’s abuse experience.

### Dissociation

Faced with re-traumatisation, dissociation was an involuntary means of self-protection for some women in which they retreated into themselves and shut the world out. The woman who had pushed her baby out in theatre with staff observing from the side of the room, described watching herself give birth from the ceiling. Another felt violated by her care and recognised the mixed messages her behaviour would have given:

*The only thing I remember is having to have loads and loads of internal examinations and being so numb to people violating me that I just didn’t really show any anxiety or embarrassment at all. People probably thought it was confidence or comfort with the professional, but it wasn’t, it’s a numb, dissociative state.* (Parent, survey)

Sometimes women were able to use dissociation to their advantage:

*And sometimes it can be really useful because I do know that I do that in lots of difficult situations, it’s learning to use it to my advantage rather than it having a control over me …* (Parent interview)

but such dissociative states can be confusing for both women and staff:

*I heard someone say to someone else, ‘she never made a sound you know’ and the other one said to her ‘well no, that’s terror’ and that was true, and I just went absolutely still. I couldn’t go anywhere; I couldn’t do anything to stop it and so I just closed down.* (Parent, focus group)

The fact that women often do not disclose their abuse to caregivers can make women’s behaviour during dissociation particularly perplexing.

### Disclosure

Few of the participants had disclosed their abuse to healthcare professionals and some had not told anyone – even their partners. Sometimes women struggled to admit to themselves that their abuse was an issue:

*I’d kind of just literally put it in a box and thought that it wouldn't impact on me too much.* (Parent, interview)

Some did not feel the need to disclose, were unwilling to do so and did not believe their history to be relevant. Others wished with hindsight that they had let someone know:

*… so I suppose I would have found it much easier if it had just been out there and so then the complexities, kind of, everything around pregnancy and childbirth and kind of sexual was sort of in the open.* (Parent, interview)

There was sometimes an assumption that everyone would just know about the woman’s experience of CSA:

*I certainly felt like everybody knew even before I told them that that had happened to me. And I haven't told many people now.* (Parent, interview)

Those who had not been pregnant were anxious about whether they should disclose their abuse and there was widespread concern as to how the information would be received. Previous experience of trying to tell people about abuse put women off disclosing:

*I had had such bad experiences of telling trusted adults in the past, that I think I projected that into the relationship with healthcare professionals.* (Parent, survey)

There was concern about whether staff would want to hear and whether it would make any difference if a history of abuse was recorded in medical records. Prior bad experiences with healthcare professionals made women cautious about disclosing. One explained that the nature of her abuse meant that her genitalia look different from other women:

*… and the one time in the past where I have been to a medical appointment that involved them looking at that, I could just tell that the person was kind of shocked and a bit like oh I don’t know what to do with this and I found that really difficult and that’s made it pretty much impossible for me to go back.* (Non-parent, focus group)

The difficulty of broaching the subject was recognised:

*At the time, I could not and did not tell the healthcare professionals of my survivor status. I did not know how to say it, and no one asked me.* (Parent, survey)

This implies that a question asked by healthcare professionals might have been helpful, but again, views were mixed. In some cases, a direct question would have been welcomed and a disclosure would have followed. In others, women acknowledged that they would deny abuse. Some women would have appreciated the opportunity to bring the subject up, even if they would not have responded:

*… it might have just put a thought in my head, even if it wasn’t something that I shared with anybody, it might have just put a thought in my head which might have been useful I think at some point.* (Parent, interview)

Being given the opportunity to discuss previous difficult experiences with healthcare professionals without necessarily naming childhood sexual abuse was an approach favoured in the focus group for non-parents. This corroborated a tactic recommended by a survey participant:

*Plus you do NOT need to tell them you are a survivor if you don't want to, but it might help if you say 'something happened to me that I prefer not to talk about which means intimacy can be an issue, so please be sensitive to that and listen to my needs….’* (Parent, survey)

Reluctance to disclose was multi-factorial, but often it was grounded in fear of judgement, particularly related to women’s ability to become a good parent.

### Parenthood

Participants who were not yet parents particularly expressed fear of judgement. There was concern that once people discovered a history of abuse there would be an assumption that they would not be good parents. Specific fears were voiced about the widespread belief that the abused become abusers:

*… because people say that people who are abused go on to abuse and that really -, I don’t know what it makes me feel, but I don’t like it at all and I do worry that people think that about me.* (Non-parent, focus group)

Women expressed apprehension about whether they would be a good mother, especially if the relationship with their own mother had been dysfunctional. Many found the reality of being a parent very difficult:

*… you're still, in some ways, stuck in your own childhood, which I can really see I have been, even though I had a job, a career, a marriage and all of those things, in some ways I was still very, very vulnerable and then suddenly you’ve got vulnerable babies relying on you and that can kind of knock you off kilter a little bit I think really.* (Parent, interview)

Having a son was a relief for some women because they believed them to be less likely to experience abuse. Those who had girls were more fearful and becoming the mother of a daughter could be unexpectedly challenging:

*Having my 2 boys I wasn't troubled by my past, but the birth of my daughter immediately brought intense feelings and memories back.* (Parent, survey)

Concern re-emerged as girls approached the age at which the mother’s abuse had begun:

*And even now, I still think now there’s something about having her that’s constantly unlocking and making stuff sort of challenging.* (Parent, interview)

Becoming a mother changed the perspective some women had on their own childhood:

*It made me very emotional to think anyone could harm a child. I was confused as to why I was harmed, and it made me fiercely protective over my daughter.* (Parent, survey)

Anxiety was not a universal experience among participants. Some reported loving being a mother and coping well with the transition required. These women particularly delighted in giving their children all that was lacking in their own childhood.

There was an overwhelming urge to protect children. Some women feared being over-protective and others recognised that they had been. Women often felt compelled to provide all care for their children themselves and could not contemplate leaving them unattended or letting them stay overnight with anyone:

*I can remember I didn’t actually want other people, apart from my husband, to really hold my babies when I first had them. I wanted them. I wanted to see them all the time. I wanted to know that they were OK, and I felt like the very best person to look after them really.* (Parent, interview)

Even when providing care themselves, women were often anxious about whether they were hurting their baby:

*She is 16 months old and I still find nappy changing tough, I don't like applying cream to her intimate area and I apologise to her all the time when I'm changing her because I don't want her to think I would ever hurt her.* (Parent, survey)

Infant feeding, specifically breastfeeding was an aspect of baby care that provoked a variety of responses. For some women, there was no question over their choice:

*I knew that I would be OK with breastfeeding because that’s exactly what they* [breasts] *are there for, so. And, you know, I have a real thing about that, … and I'm a bit of an earth mum, that for me was the only consideration really.* (Parent, interview)

Others, especially if their abuse had involved their breasts, were more ambivalent. Some struggled with the sensation of breastfeeding and some made a conscious decision to use bottles from the start. For those contemplating breastfeeding, potential exposure of their breasts was worrying:

*I wouldn’t want to openly breastfeed where people could see … because I don’t want to expose my body ever again.* (Non-parent, focus group)

Those who had breastfed had generally navigated their way round that issue. They had worked out how to feed without having to ‘show any cleavage or anything like that … ’ (Parent, interview). As with other aspects of the journey to parenthood, intervention from family, friends or healthcare professionals was as much of a concern as feeding itself:

*I think I would feel fine about breastfeeding my baby, I don’t think that would be my issue, I think the issue would come in if like, you know, you're having midwives or you're having relatives or friends being like ‘oh you need to do it like this, oh no you're not doing it quite right, let me show you’ - and then suddenly it’s something that should be quite private and intimate is being taken … .* (Non-parent, focus group)

Fear of judgement was universal, but the women’s experiences were individual and varied.

The final resource was uploaded to TST website (https://www.thesurvivorstrust.org/pbpaftercsa. Additional file 1 shows the e-flyer). It is accessible by mobile phone, tablet, laptop and desktop computers. A link to an evaluation form was included at the end of the resource. Since its launch in June 2019, the opening page has had 3114 views and 2607 (84%) have accessed the resource. The average time spent on the resource was 7 min. However, those accessing the resource have not engaged with the request for evaluation. The landing page for the resource was therefore amended to highlight the request for evaluation and a link to the evaluation form provided.

## Discussion

The co-production process for this resource ensured that it is grounded in survivors’ voices, experiences and expertise [11]. As such, it is consistent both with participatory research and trauma-informed principles [20]. Importantly, it speaks with women’s words in a peer-to-peer voice. As a researcher who had previously investigated the maternity care experiences of women who have experienced CSA, the first author had insight into potential information needs that could be addressed by the resource. The risk of bias was minimised by our initial facilitative approach that explored the views of those with lived experience of abuse on which the outline plan was then based.

As well as informing the development of the resource, data collected during Phase 1 of the study adds to the body of evidence on the maternity care experiences and needs of those who have experienced CSA. The findings corroborated previous qualitative work on maternity care experiences of women who have experienced CSA. This includes responses to pregnancy [7, 21–23] and concern about physical damage from the abuse [7]. In common with previous work, re-living abuse was a significant theme in our study. It was one of three phases for ‘moving beyond the pain’ in Roller’s study [24] in which triggers included invasive, intimate procedures like vaginal examinations but also less predictably difficult sensations of pregnancy such as fetal movements. Fetal movements are a tangible manifestation that just as during childhood, a woman’s body has been taken over by someone else. Pregnancy itself can therefore be triggering and challenge a woman’s integrity [7] as it was for the woman in our study who felt ambivalent about letting go of her body again having reclaimed it following abuse. Triggers were a concern for those who had not yet had children. Those who had, acknowledged the unpredictability of them. By their nature triggers are individual, but they can also take people by surprise [7]. One of the sections in the resource therefore provides examples of the sort of things that have been triggering for others so that users could think through what might be difficult for them. Lack of control is reminiscent of abuse for many and is a pre-eminent theme within the literature [8, 10, 21–24]. The consequent re-traumatisation can leave women feeling very vulnerable. Alternatively being in control is a key aspect of feeling safe [10] which is important as women face pregnancy and birth. There is a section on control in the resource which shares women’s experiences of both control and lack of control. It also provides suggestions for regaining control that our co-producers have found helpful.

### Strengths and limitations

The resource that was developed, produced, and launched following the processes reported in this paper was co-produced by women with lived experience of CSA. Their intrinsic input throughout each stage of the work increases its credibility. Corroboration of key themes from our Phase 1 data in the wider literature, suggest that the content of the resource is valid information.

However, there were limitations to the project. It is challenging to engage survivors and a number of women who planned to attend focus groups and workshops in person did not feel able to travel on the day. Some of these participated via the electronic options, but not all did. The smaller number of participants in Phase 2 requires the development team to remain vigilant to feedback. The work was designed for use within the UK and links to the NHS provide a considerable amount of the supporting information. The resource as a whole may not therefore be transferable to settings outside of the UK, but the voices of the women, heard through film, animation and written quotations may be relevant.

We recognise that ‘survivor’ is a contested term and new options may emerge as talking about sexual abuse becomes less taboo and new vocabulary takes root in popular culture that can inform healthcare discourse.

## Conclusions

This paper describes the development of an innovative and accessible e-resource for those who have experienced CSA and are contemplating pregnancy, birth, and parenthood. It is based on the words and experiences of survivors. A range of experience has been presented to reflect individuality and to convey the message that there is no ‘right’ way to approach pregnancy and birth. The resource recognises the importance of control and feeling safe. We hope that those who use the resource will feel empowered as they embark on pregnancy, birth, and parenthood after childhood sexual abuse.

## Data Availability

Qualitative data extracts are presented in the article to support the findings. The original transcripts are not available to the public as they may contain information that could compromise the confidentiality and anonymity of the participants.

## Abbreviations

CSA: Childhood Sexual Abuse
TST: The Survivors Trust

## References

[1] Pereda N, Guilera G, Forns M, Gómez-Benito J (2009). The international epidemiology of child sexual abuse: a continuation of Finkelhor (1994). Child Abuse Negl.

[2] Elkin M (2020). Child sexual abuse in England and Wales : year ending March 2019.

[3] Pan Y, Lin X, Liu J, Zhang S, Zeng X, Chen F, et al. Prevalence of childhood sexual abuse among women using the childhood trauma questionnaire: a worldwide meta-analysis. Trauma Violence Abuse. 2020.p. 1–11. 10.1177/1524838020912867. 10.1177/1524838020912867 32207395

[4] Children’s Commissioner. Protecting children from harm: a critical assessment of child sexual abuse in the family network in England and priorities for action. London: Children’s Commissioner for England; 2015.

[5] Itzin C, Taket A, Barter-Godfrey S (2010). Domestic and sexual violence and abuse: tackling the health and mental health effects.

[6] American Psychiatric Association. Diagnostic and statistical manual of mental disorders. 5th ed. Washington DC: APA; 2013.

[7] Montgomery E, Pope C, Rogers J. The re-enactment of childhood sexual abuse in maternity care: a qualitative study. BMC Pregnancy Childbirth. 2015;15. 10.1186/s12884-015-0626-9. 10.1186/s12884-015-0626-9 PMC4549944 26306798

[8] Montgomery E, Pope C, Rogers J. The re-enactment of childhood sexual abuse in maternity care: a qualitative study. BMC Pregnancy Childbirth. 2015;15:194:54-60. 10.1186/s12884-015-0626-9 PMC4549944 26306798

[9] Montgomery E. Voicing the silence: the maternity care experiences of women who were sexually abused in childhood. University of Southampton; 2012. 25958452

[10] Montgomery E (2013). Feeling safe: a Metasynthesis of the maternity care needs of women who were sexually abused in childhood. Birth..

[11] Perot C, Chevous J, Survivors’ voices research group (2018). Turning pain into power a charter for organisations engaging abuse survivors in projects, research & service development.

[12] Bogart LM, Uyeda K (2009). Community-based participatory research: partnering with communities for effective and sustainable behavioral health interventions. Health Psychol.

[13] Cargo M, Mercer SL (2008). The value and challenges of participatory research: strengthening its practice. Annu Rev Public Health.

[14] Harvey M, Land L (2017). Research methods for nurses and midwives: theory and practice.

[15] Bergold J, Thomas S (2012). Participatory research methods: a methodological approach in motion.

[16] Wilkinson S. Focus group research. In: Silverman D, editor. Qualitative Research: Theory, method and practice. 2nd ed. London: Sage; 2004.

[17] Walsh D, Baker L, Lavender T, Edwards G, Alfirevic Z (2004). How to collect qualitative data. Demystifying qualitative research.

[18] Kitzinger J, Barbour R, Barbour R, Kitzinger J (1999). The challenge and promise of focus groups. Developing focus group research.

[19] Braun V, Clarke V (2006). Using thematic analysis in psychology. Qual Res Psychol.

[20] Substance Abuse and Mental Health Services Administration. SAMHSA’s Concept of Trauma and Guidance for a Trauma-Informed Approach. HHS Publication No. (SMA) 14-4884. Rockville, MD: Substance Abuse and Mental Health Services Administration; 2014.

[21] LoGiudice JA, Beck CT (2016). The lived experience of childbearing from survivors of sexual abuse: “it was the best of times, it was the worst of times.”. J Midwifery Womens Health.

[22] Sperlich M, Seng JS (2008). Survivor moms: Women’s stories of birthing, mothering and healing ater sexual abuse.

[23] Seng JS, Sparbel KJH, Low LK, Killion C (2002). Abuse-related posttraumatic stress and desired maternity care practices: women’s perspective. J Midwifery Womens Health.

[24] Roller CG (2011). Moving beyond the pain: women’s responses to the perinatal period after childhood sexual Abuse. J Midwifery Womens Health.

